# Prevalence of Sleep-Disordered Breathing and Cheyne-Stokes Respiration in Patients With Atrial Fibrillation

**DOI:** 10.31083/RCM46143

**Published:** 2025-08-29

**Authors:** Hiroki Matsumoto, Takatoshi Kasai, Akihiro Sato, Nanako Shiroshita, Sayaki Ishiwata, Shoichiro Yatsu, Jun Shitara, Takao Kato, Shoko Suda, Ryo Naito, Hidemori Hayashi, Tohru Minamino, Hiroyuki Daida

**Affiliations:** ^1^Department of Cardiovascular Biology and Medicine, Juntendo University Graduate School of Medicine, 113-8421 Tokyo, Japan; ^2^Cardiovascular Respiratory Sleep Medicine, Juntendo University Graduate School of Medicine, 113-8421 Tokyo, Japan; ^3^Sleep and Sleep-Disordered Breathing Center, Juntendo University Hospital, 113-8421 Tokyo, Japan; ^4^Department of Cardiovascular Management and Remote Monitoring, Juntendo University Graduate School of Medicine, 113-8421 Tokyo, Japan

**Keywords:** atrial fibrillation, sleep-disordered breathing, Cheyne-Stokes respiration

## Abstract

**Background:**

Limited data are available regarding the prevalence of sleep-disordered breathing (SDB), particularly Cheyne–Stokes respiration (CSR), in patients with atrial fibrillation (AF) and left ventricular (LV) systolic dysfunction. Thus, this study aimed to investigate the prevalence of SDB and CSR, as well as the factors associated with these conditions, in patients with AF without LV systolic dysfunction.

**Methods:**

Patients with paroxysmal and non-paroxysmal AF underwent echocardiography and cardiorespiratory polygraphy. Multiple linear regression analysis was performed using the apnea–hypopnea index (AHI) and %CSR as the dependent variables.

**Results:**

A total of 462 patients were enrolled; 335 patients (72.5%) were diagnosed with SDB (AHI ≥5/h), with a median AHI of 10.3 events per hour (interquartile range, 4.7–20.8). CSR was observed in 107 patients (23.2%). Multiple linear regression analysis showed that age, sex, body mass index, and hypertension were independently correlated with AHI (*p* = 0.0188, 0.0002, <0.0001, and 0.0457, respectively). Conversely, age, diabetes mellitus (DM), and the plasma N-terminal prohormone of brain natriuretic peptide (NT-proBNP) level were independently correlated with %CSR (*p* < 0.0001, 0.0047, and 0.0095, respectively).

**Conclusion:**

SDB and CSR were common in patients with AF. CSR was observed in older patients with DM and high NT-proBNP levels.

## 1. Introduction

Atrial fibrillation (AF) affects approximately 2.5% of adults above 40 years [1] and is associated with an increased risk of ischemic stroke and heart failure (HF) [2, 3]. In general, diabetes mellitus (DM), hypertension, obesity, alcohol consumption, and sleep-disordered breathing (SDB) are important modifiable risk factors for AF [4]. In patients with obstructive sleep apnea (OSA), several pathophysiologic mechanisms, such as exaggerated negative intrathoracic pressure, sympathetic overactivity, and intermittent nocturnal hypoxia/reoxygenation, contribute to cardiac overload, increased left ventricular (LV) filling pressure, and electrical and structural remodeling of the atrium, all of which predispose to the development of an AF substrate [5, 6, 7, 8]. Autonomic dysregulation associated with each respiratory event has been linked to the development of incident AF [6]. Indeed, the prevalence of SDB has been reported as 40–57% in patients with AF [8]. Since patients with AF and SDB often do not report excessive daytime sleepiness or fatigue [9], and access to sleep testing is limited, SDB may be underdiagnosed in patients with AF [10]. Thus, knowledge regarding the prevalence and associated factors of SDB in patients with AF is needed.

Cheyne-Stokes respiration (CSR), characterized by repetitive apneas or hypopneas alternating with hyperventilation in a crescendo-decrescendo pattern of tidal volume [11], is generally associated with HF through pulmonary congestion and prolonged circulation time in association with low cardiac output [8]. Although several studies have reported on the prevalence of CSR in patients with LV systolic dysfunction—regardless of the presence or absence of AF [8, 12]—and while an association between AF and CSR has been suggested in HF patients with LV systolic dysfunction [12], specific data regarding CSR in AF patients without LV systolic dysfunction are unavailable. Furthermore, data regarding the correlates of CSR in this patient population remain unclear. Thus, in the present study, we investigated the prevalence of SDB and CSR, and factors associated with them in patients with AF without LV systolic dysfunction.

## 2. Methods

### 2.1 Participants

Patients with paroxysmal or non-paroxysmal AF without LV systolic dysfunction were prospectively enrolled at Juntendo University Hospital between May 2017 and March 2024. Paroxysmal AF was defined as an AF episode that terminated within seven days of onset, either spontaneously or after the administration of antiarrhythmic drugs. Non-paroxysmal AF was defined as an AF episode lasting for more than seven days [13]. Patients with a previous diagnosis or treatment of SDB, LV ejection fraction (LVEF) ≤45% on echocardiogram, severe organic valvular heart disease, life-threatening diseases (uncured malignancy, severe chronic pulmonary diseases with oxygen inhalation, and end-stage renal disease with dialysis), and pregnancy were excluded. The Institutional Review Board of Juntendo University Hospital approved the study protocol, which complied with the Declaration of Helsinki. Informed consent was obtained from all patients.

### 2.2 Sleep Study

For the sleep study, all patients underwent cardiorespiratory polygraphy (ApneaLink Air; ResMed, Sydney, Australia). CSR determined by ApneaLink is clinically acceptable, as it has been used in large-scale studies [14, 15, 16] and because performing polysomnography in all AF patients is not considered feasible [10]. Thus, we opted to use ApneaLink. Respiratory effort, airflow measured via the pressure sensor, snoring, pulse, oxygen saturation, and percentage of CSR patterns were recorded. This device provides a flow-based classifier for CSR. Apnea was defined as a ≥80% decrease in airflow for ≥10 seconds; hypopnea was defined as a decrease in airflow by ≥50–80% versus baseline for ≥10 seconds; and desaturation was defined as a ≥4% decrease in oxygen saturation. Patients were diagnosed with SDB when the apnea-hypopnea index (AHI) was >5/h, and they were divided into mild (AHI ≥5/h), moderate (15 ≤ AHI < 30/h), and severe (AHI ≥30/h) SDB groups. CSR was identified based on the cycle length (usually 45–90 s), apnea-hypopnea length, hyperpnea length, and shape of the hyperpnea, regardless of types of respiratory events. The typical waxing and waning shapes of a flow can be mathematically described as a linear combination of various trigonometric functions [14]. Because the original description of CSR does not specify the type of respiratory events and instead focuses on the form of hyperventilation observed [11], CSR which does not specify the type of respiratory events (i.e., central or obstructive) is of our interest. %CSR is the percentage of the length of CSR divided by the flow evaluation time and is used in large-scale registries or a randomized controlled study involving CSR [16]. In this study, coexisting CSR was defined as %CSR >0%. Since no specific thresholds for %CSR have been established, the prevalences of cases with %CSR >10% and >20% were also reported.

### 2.3 Data Collection

Demographic and medical histories were obtained through clinical chart reviews and interviews. Blood tests and echocardiographic data were obtained within one month of the sleep study. Serum levels of N-terminal prohormone of brain natriuretic peptide (NT-proBNP) were measured, and the estimated glomerular filtration rate (eGFR) was calculated using the Modification of Diet in Renal Disease equation with a Japanese coefficient [17]. Complete two-dimensional echocardiography was performed according to the American Society of Echocardiography guidelines [18]. Images were stored for at least three cardiac cycles, and the final values represent the average of at least three measurements. LVEF was calculated using the modified Simpson method. All echocardiographic studies were performed and interpreted by experienced cardiologists who were blinded to the clinical data.

### 2.4 Statistical Analysis

Continuous variables are expressed as mean ± standard deviation (SD) or median (interquartile range). Categorical data are presented as frequencies and ratios (%). The characteristics and echocardiographic data of patients with and without CSR were compared using the chi-square or Fisher’s exact test for categorical variables and the *t*-test or Mann-Whitney U test for continuous variables. In addition, NT-proBNP levels, AHI, body mass index (BMI), and %CSR were compared between patients with paroxysmal and non-paroxysmal AF. Univariable and multivariable linear regression analyses, including AHI as a dependent variable, were performed. In addition, univariable and multivariable linear regression analyses, including %CSR as a dependent variable, were performed. Because the %CSR data were skewed and contained zero values, we applied natural log transformation using the formula: Ln %CSR = log (%CSR + 0.01) [19]. The multivariable analysis included independent variables with a significance of <0.05 from the univariate analyses to determine the factors associated with AHI or %CSR. Statistical significance was set at *p* < 0.05. All analyses were performed using JMP Pro version 18 (SAS Institute Inc., Cary, NC, USA).

## 3. Results

### 3.1 Prevalence of SDB

**Fig. 1.**
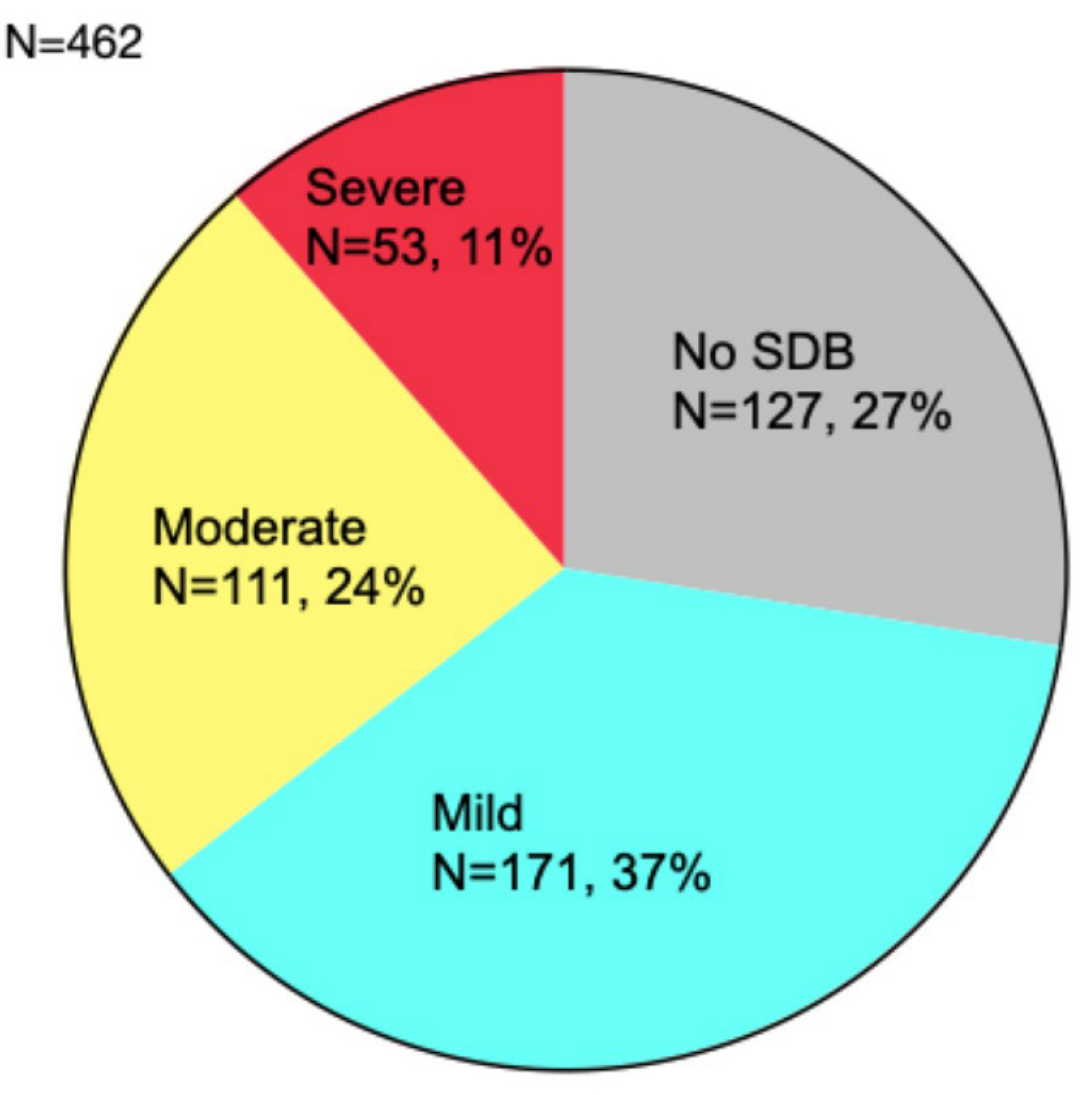
**Severity and prevalence of SDB**. SDB was observed in 335 patients (72.5%). SDB, sleep-disordered breathing.

**Fig. 2.**
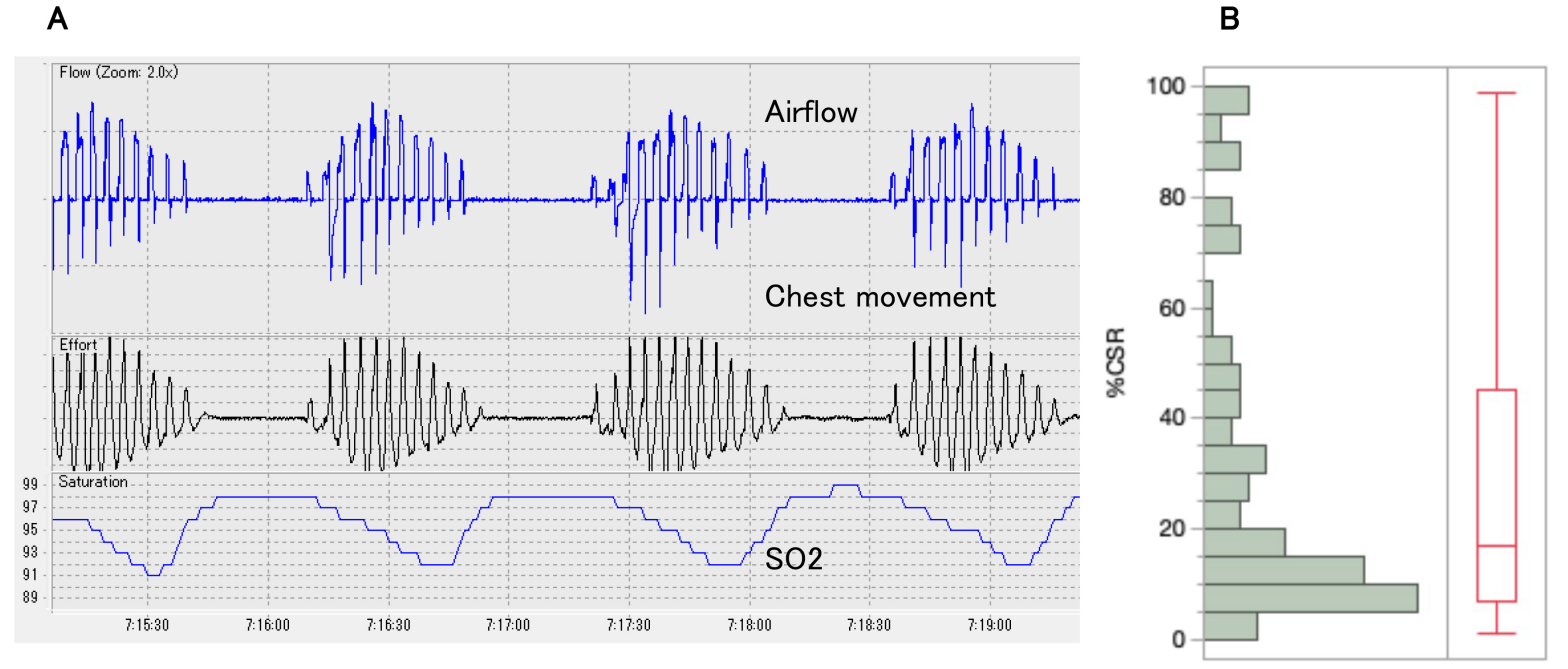
**CSR observed in this study**. (A) Typical CSR pattern: CSR is characterized by repetitive central apneas alternating with hyperventilation in a crescendo-decrescendo tidal volume pattern. (B) Distribution of %CSR: In patients with CSR (n = 107), the median %CSR was 17% (interquartile range, 7–45). CSR, Cheyne-Stokes respiration.

**Fig. 3.**
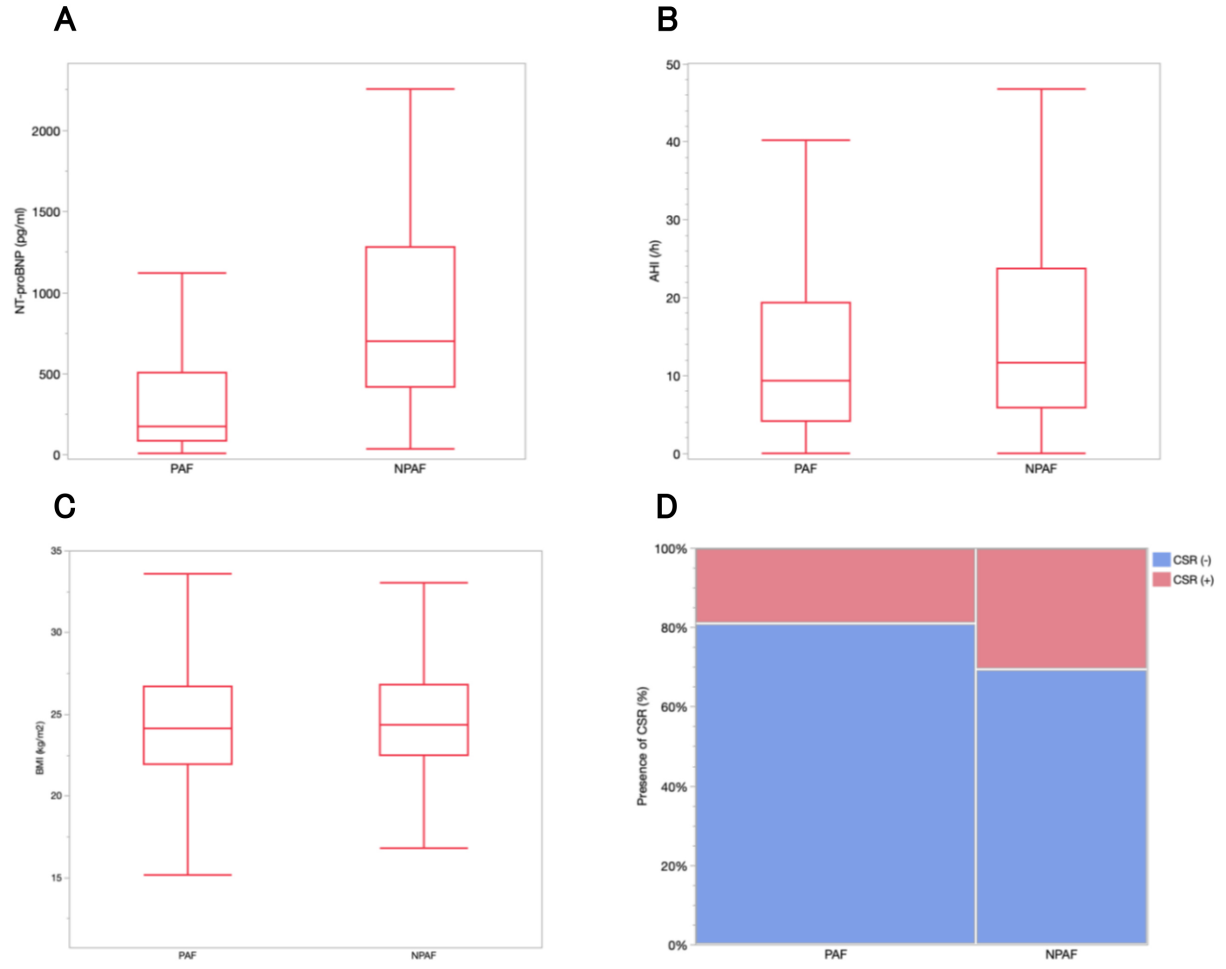
**NT-proBNP levels, AHI, and the percentage of patients with CSR according to AF type**. (A) The median NT-proBNP level was 176 pg/mL (interquartile range, 83.8–504.5) in patients with PAF and 700.5 pg/mL (interquartile range, 414–1279.3) in those with NPAF (*p* < 0.0001). (B) The median AHI was 9.25 events per hour (interquartile range, 4.1–19.3) in patients with PAF and 11.6 events per hour (interquartile range, 5.9–23.7) in those with NPAF (*p* = 0.0142). (C) The median BMI was 24.1 (interquartile range, 21.9–26.7) in patients with PAF and 24.3 (interquartile range, 22.5–26.8) in those with NPAF (*p* = 0.2085). (D) The percentage of patients with CSR was 54 (19.0%) with PAF and 53 (30.5%) with NPAF (*p* = 0.0047). AHI, apnea-hypopnea index; PAF, paroxysmal atrial fibrillation; NPAF, non-paroxysmal atrial fibrillation; CSR, Cheyne-Stokes respiration; BMI, body mass index; NT-proBNP, N-terminal prohormone of brain natriuretic peptide.

A total of 462 patients underwent cardiorespiratory polygraphy; 37% had mild (AHI ≥5/h), 24% had moderate (15 ≤ AHI < 30/h), and 11% had severe (AHI ≥30/h) SDB (Fig. 1). The median AHI was 10.3 (4.7–20.8)/h. CSR was identified in 107 patients (23.2%). The prevalences of %CSR >10% and >20% were 15.4% (71/462) and 10.8% (50/462), respectively. Typical CSR patterns and the distribution of %CSR in patients with CSR are shown in Fig. 2. Patient characteristics and echocardiography data are shown in Tables 1,2. The cardiorespiratory polygraphy data are shown in Table 3. NT-proBNP levels, AHI, and CSR percentages in patients with paroxysmal and non-paroxysmal AF are shown in Fig. 3.

**Table 1.** **Patient characteristics**.

Characteristic	All N = 462	CSR (+) N = 107	CSR (-) N = 355	*p*-value
Age, years	67.6 ± 11.3	73.1 ± 8.3	65.9 ± 11.2	<0.0001
Female sex, n (%)	104 (23%)	20 (19%)	84 (24%)	0.2747
BMI, kg/m^2^	24.6 ± 3.8	24.8 ± 3.8	24.5 ± 3.8	0.4968
Non-paroxysmal AF, n (%)	175 (38%)	53 (50%)	122 (34%)	0.0049
History of HF, n (%)	111 (24%)	40 (37%)	71 (20%)	0.0002
History of stroke, n (%)	39 (8%)	9 (8%)	30 (9%)	0.9711
Diabetes mellitus, n (%)	96 (21%)	35 (33%)	61 (17%)	0.0005
Hypertension, n (%)	252 (55%)	66 (62%)	186 (53%)	0.0873
Dyslipidemia, n (%)	199 (43%)	43 (41%)	156 (44%)	0.4945
Hyperuricemia, n (%)	117 (25%)	30 (28%)	87 (25%)	0.4580
Warfarin, n (%)	43 (9%)	14 (13%)	29 (8%)	0.1241
DOAC, n (%)	385 (84%)	90 (85%)	295 (84%)	0.7864
ACE/ARB/ARNI, n (%)	189 (41%)	56 (53%)	133 (38%)	0.0058
Beta blocker, n (%)	260 (57%)	77 (73%)	183 (52%)	0.0002
CCB, n (%)	170 (37%)	38 (35%)	132 (38%)	0.7577
Diuretics, n (%)	101 (22%)	38 (36%)	63 (18%)	<0.0001
Statin, n (%)	126 (27%)	37 (35%)	89 (25%)	0.0518
Antiarrhythmic drug, n (%)	197 (43%)	38 (36%)	159 (45%)	0.0890
Hemoglobin, g/dL	14.3 ± 1.7	14.4 ± 1.8	14.3 ± 1.7	0.6326
Creatinine, mg/dL	0.9 ± 0.3	1.0 ± 0.3	0.9 ± 0.3	0.0001
eGFR, mL/min/1.73 cm^2^	66.4 ± 17.5	59.7 ± 17.4	68.3 ± 17.0	<0.0001
NT-proBNP, pg/mL	342 (134.0–810.8)	698 (284.5–1253.3)	269 (116.5–677)	<0.0001

Data are expressed as mean ± SD or median (interquartile range) for continuous variables and numbers (%) for nominal variables. AF, atrial fibrillation; ACE, angiotensin-converting enzyme; ARB, angiotensin II receptor blocker; ARNI, angiotensin receptor neprilysin inhibitor; BMI, body mass index; CCB, calcium channel blocker; DOAC, direct oral anticoagulant; eGFR, estimated glomerular filtration rate; HF, heart failure; NT-proBNP, N-terminal prohormone of brain natriuretic peptide.

**Table 2.** **Echocardiography data**.

Variable	N = 462	CSR (+) N = 107	CSR (-) N = 355	*p*-value
LVDd, mm	47.3 ± 5.6	48.0 ± 5.8	47.2 ± 5.4	0.1778
LVDs, mm	31.1 ± 6.2	32.4 ± 6.9	30.8 ± 5.9	0.0150
IVSd, mm	9.8 ± 1.6	10.3 ± 1.4	9.7 ± 1.7	0.0002
PWd, mm	9.7 ± 1.3	10.2 ± 1.3	9.6 ± 1.3	<0.0001
LVEF, %	62.9 ± 10.1	60.6 ± 11.6	63.6 ± 9.5	0.0055
LAVI, mL/m^2^	45.5 ± 23.0	54.7 ± 30.0	42.7 ± 19.6	<0.0001
DcT, ms	186.4 ± 60.3	187.6 ± 67.7	186.3 ± 58.0	0.8499
E/e’	10.6 ± 5.3	11.7 ± 5.2	10.3 ± 5.3	0.0162
RVSP, mmHg	25.7 ± 7.2	27.0 ± 7.9	25.2 ± 7.0	0.0264

Data are expressed as mean ± SD for continuous variables. DcT, deceleration time; IVSd, interventricular septum (diastolic); LAVI, left atrial volume index; LVDd, left ventricular diameter (diastolic); LVDs, left ventricular diameter (systolic); PWd, posterior wall (diastolic); RVSP, right ventricular systolic pressure; LVEF, left ventricular ejection fraction.

**Table 3.** **Sleep polygraphy data**.

Variable	N = 462
AHI (events per hour)	10.3 (4.7–20.8)
3% ODI (events per hour)	13.5 (7.0–24.7)
Mean SpO_2_ (%)	95.0 (94.0–96.0)
Lowest SpO_2_ (%)	85.0 (80.0–89.0)
Coexisting CSR, n (%)	107 (23.2%)

Data are expressed as median (interquartile range) for continuous variables and numbers (%) for nominal variables. AHI, apnea-hypopnea index; CSR, Cheyne-Stokes respiration; ODI, oxygen desaturation index; SpO_2_, oxyhemoglobin saturation.

### 3.2 Factors Associated With AHI and %CSR

Univariate analysis showed that age, sex, BMI, hypertension, left atrial volume index (LAVI), and non-paroxysmal AF were correlated with AHI. Multivariate analysis identified age, sex, BMI, and hypertension as independent correlates of AHI (Table 4).

**Table 4.** **Univariable and multivariable linear regression analysis for AHI**.

	Univariable		Multivariable	
	Correlation coefficient	*p*	Partial correlation coefficient	*p*
Age	0.095	0.0406	0.122	0.0188
Female sex	–0.174	0.0002	–0.181	0.0002
BMI	0.250	<0.0001	0.230	<0.0001
Hypertension	0.198	<0.0001	0.099	0.0457
LAVI	0.095	0.0479	0.055	0.2889
non-paroxysmal AF	0.098	0.0362	–0.024	0.6296

BMI, body mass index; LAVI, left atrial volume index; AF, atrial fibrillation.

Univariate analysis showed that age, DM, angiotensin-converting enzyme (ACE)/angiotensin II receptor blocker (ARB)/angiotensin receptor neprilysin inhibitor (ARNI), beta-blockers, diuretics, eGFR, NT-proBNP, LVEF, and E/e’ were associated with %CSR. Multivariate analysis identified age, DM, and NT-proBNP level as independent correlates of %CSR (Table 5).

**Table 5.** **Univariable and multivariable linear regression analysis of CSR**.

	Univariable		Multivariable	
	Correlation coefficient	*p*	Partial correlation coefficient	*p*
Age	0.284	<0.0001	0.246	<0.0001
DM	0.159	0.0006	0.138	0.0047
ACE/ARB/ARNI	0.153	0.0011	0.053	0.3263
Beta-blocker	0.179	0.0001	0.086	0.0995
Diuretics	0.211	<0.0001	0.058	0.3297
eGFR	0.221	<0.0001	0.025	0.6830
NT-proBNP	0.242	<0.0001	0.152	0.0095
LVEF	0.150	0.0013	0.043	0.4475
E/e’	0.121	0.0115	0.031	0.5892

DM, diabetes mellitus; ACE, angiotensin-converting enzyme; ARB, angiotensin II receptor blocker; ARNI, angiotensin receptor neprilysin inhibitor; eGFR, estimated glomerular filtration rate; NT-proBNP, N-terminal prohormone of brain natriuretic peptide; LVEF, left ventricular ejection fraction.

## 4. Discussion

The findings of this study were as follows: (1) 72.5% of AF patients had SDB; (2) 23.1% of AF patients had CSR; (3) AHI was associated with age, sex, BMI, and hypertension; and (4) the presence of CSR was associated with older age, DM, and elevated NT-proBNP levels, suggesting that in patients with AF, SDB is highly prevalent, particularly among older, obese men with hypertension. CSR is also prevalent, particularly among older patients with diabetes and high LV filling pressure. Since specific data regarding CSR in AF patients without LV systolic dysfunction are lacking, the finding that 23% of these patients have CSR—along with the abovementioned correlates that are similar to those seen in other patient populations—represents a novel and significant contribution of the present study. The correlates of AHI and CSR identified in the present study are plausible. In the general population, factors such as age, male sex, BMI, and comorbid hypertension are associated with increased AHI. Additionally, in other patient populations, older patients with elevated LV filling pressure are more likely to exhibit CSR. Moreover, limited data suggest that patients with DM may experience CSR, even in the absence of AF [20, 21].

In terms of prevalence of SDB, Traaen *et al*. [22] found that 82.7% of 579 patients with paroxysmal AF had SDB (AHI ≥5/h, by polygraphy), and age, male sex, and BMI were correlates of SDB. Tanaka *et al*. [23] studied polygraphy in 776 patients with AF; 88% were diagnosed with SDB (AHI ≥5/h), and 53.2% had moderate-to-severe SDB (AHI ≥15/h). In their study, obesity, male sex, non-paroxysmal AF, hypertension, and left atrial dilatation were associated with moderate-to-severe SDB. Similarly, the present study confirmed a high prevalence of SDB even among AF patients without LV systolic dysfunction. This elevated prevalence of SDB in AF patients may be partly explained by the cause-and-effect relationship between AF and SDB, especially OSA. Atrial stretching following negative thoracic pressure, along with autonomic dysregulation, contributes to electrical remodeling in patients with OSA [24]. Indeed, prolongation of P-wave duration in electrocardiogram, which is suggestive of atrial dilatation and its relationship with arterial stiffness, was shown in patients with OSA [7]. In patients with paroxysmal AF, OSA was associated with lower voltage amplitude in the atria and increased incidence of non-pulmonary vein triggers [25]. Chronic intermittent hypoxia increases inflammatory cytokine levels, and oxidative stress facilitates structural remodeling [26, 27]. Under these mechanisms, an AF substrate is formed, contributing to the development of AF in patients with OSA. Thus, OSA is regarded as a cause of AF, and it is plausible that AHI correlates are similar to those observed in the general population.

However, the relationship between AF and CSR may be bidirectional. On one hand, CSR can contribute to the development of AF. Previous studies have reported that central sleep apnea as suggestive of CSR is a predictor of incident AF, even in cohort studies [28, 29]. For instance, Tung *et al*. [30] reported that central sleep apnea as suggestives of CSR was associated with approximately a twofold increase in the odds of developing AF. Similarly, May *et al*. [31] reported that central sleep apnea as suggestives of CSR predicted a higher risk of AF in older men, particularly those aged 76 years or older. In patients with HF, one of the risk factors for CSR with central sleep apnea is AF, alongside male sex, age >60 years, and hypocapnia [32]. Although no specific data regarding relationship between CSR and incident AF is available, CSR causes periodic hypoxia and arousal, leading to autonomic alterations that may be associated with incident AF [12, 33]. On the other hand, AF is a cause of CSR; it may promote or worsen CSR. Hypocapnia due to pulmonary congestion, increased chemosensitivity, and prolonged circulation time are all key factors in the pathogenesis of CSR [12]. Both persistent/permanent and paroxysmal AF can worsen pulmonary congestion [34, 35, 36] and prolong circulation time [37], leading to CSR. This is supported by our current findings showing an association between CSR and parameters suggestive of elevated LV filling pressure. However, to better understand the underlying relationship between AF and CSR, assessments of carbon dioxide (CO_2_) level, chemosensitivity, and direct measurements of pulmonary hemodynamics are needed.

In the present study, older age was significantly associated with CSR. This aligns with previous reports indicating that central sleep apnea or CSR is more prevalent in older adults [12, 29, 30, 38], and this is true in AF patients without LV systolic dysfunction. Although the reasons for this age-related increase remain unclear, age-related diastolic dysfunction [39], which is associated with increased LV filling pressure, may partly explain this finding in our patient population. Our findings also suggest that DM is associated with an increased likelihood of CSR in patients with AF. This may be due to diabetes-associated autonomic dysfunction, which enhances chemoreceptor sensitivity [20]. Additionally, data from the Sleep Heart Health Study showed that periodic breathing patterns were more common in patients with DM [21]. Although the other CSR correlates found in the present study are similar to those in other patient populations, physicians should consider the relationship between CSR and DM when managing patients with AF.

A recent randomized controlled trial showed that short-term continuous positive airway pressure (CPAP) therapy did not significantly reduce AF burden in patients with paroxysmal AF and moderate-to-severe SDB, probably due to insufficient statistical power and a short follow-up period [40]. However, several observational studies have indicated that CPAP therapy for SDB may be beneficial for rhythm control in AF [41]. Thus, CPAP is generally considered a treatment option for managing SDB in patients with AF. Nevertheless, clinicians encounter patients with AF whose SDB cannot be sufficiently alleviated by CPAP. Considering the findings of the present study, the presence of CSR may explain why certain cases of SDB are resistant to CPAP in patients with AF. Furthermore, in patients with AF, SDB assessments are likely to be performed using polygraphy, which cannot detect CSR, contributing to this challenge. Although not specific to patients with AF, unsuppressed SDB by CPAP is associated with poor prognosis in patients with HF and systolic dysfunction [42, 43]. Thus, the therapeutic options for SDB in patients with AF require further discussion.

The main limitation of this study is its observational, single-academic center design, and the findings are based on a cross-sectional analysis. Therefore, the results do not support a cause-and-effect relationship between SDB or CSR and other factors. Furthermore, sleep studies were conducted using polygraphy, rather than polysomnography. Accurate diagnosis of CSR and differentiation of respiratory events ideally requires polysomnography with monitoring of the CO_2_ level or ventilation data. However, polysomnography is infeasible for all patients with AF because of the associated costs, limited access, and long waiting times [10]. Therefore, in the present study, we evaluated SDB and CSR using polygraphy, which is a simple, inexpensive, and feasible tool. Nevertheless, the absence of multi-night polygraphy recordings represents a limitation. Additionally, we did not perform systematic assessments of CSR before and after AF treatment, and electrocardiogram was not included in the polygraphy of this study. Therefore, the relationship between CSR and AF burden remains unclear. Finally, the specific effects of coexisting SDB or CSR on clinical outcomes, including incident HF or stroke, are still unknown in this patient population. Further studies are needed to investigate the effects of coexisting SDB and CSR on the clinical outcomes of patients with AF.

## 5. Conclusion

SDB is common in patients with AF, and its severity is associated with aging, male sex, increased BMI, and comorbid hypertension. CSR is also common and is linked to aging, DM, and increased NT-proBNP levels, suggesting the presence of pulmonary congestion.

## Availability of Data and Materials

The datasets used and analyzed during the current study are available from the corresponding author upon reasonable request.
